# Predictors of complementary feeding practices in Afghanistan: Analysis of the 2015 Demographic and Health Survey

**DOI:** 10.1111/mcn.12696

**Published:** 2018-11-29

**Authors:** Muzi Na, Víctor M. Aguayo, Mary Arimond, Piyali Mustaphi, Christine P. Stewart

**Affiliations:** ^1^ Department of Nutritional Sciences, College of Health and Human Development Pennsylvania State University State College Pennsylvania; ^2^ Nutrition Section, Programme Division United Nations Children's Fund (UNICEF) New York New York; ^3^ Intake–Center for Dietary Assessment FHI 360 Washington DC; ^4^ Nutrition Section United Nations Children's Fund (UNICEF) Kabul Afghanistan; ^5^ Program in International and Community Nutrition, Department of Nutrition University of California Davis California

**Keywords:** Afghanistan, complementary feeding, Demographic and Health Survey, multi‐level models

## Abstract

Despite improvements over the past 20 years, high burdens of child mortality and undernutrition still coexist in Afghanistan. Global evidence indicates that complementary feeding (CF) practices predict child survival and nutritional status. Our study aims to describe CF practices in Afghanistan and to discern underlying predictors of CF by analysing data from Afghanistan's 2015 Demographic and Healthy Survey. Multilevel models were constructed comprising potential predictors at individual, household, and community levels and four CF indicators: timely introduction of solid, semi‐solid, or soft foods (INTRO), minimum meal frequency (MMF), minimum dietary diversity (MDD), and minimum acceptable diet (MAD) among breastfed children. INTRO prevalence among children aged 6–8 months was 56%, whereas the prevalence of MMF, MDD, and MAD among children aged 6–23 months was 55%, 23%, and 18%, respectively. Of the seven food groups considered, four were consumed by 20% or fewer children: eggs (20%), legumes and nuts (18%), fruits and vegetables (15%), and flesh foods (14%). Increasing child age and more antenatal care visits were significantly and positively associated with greater odds of meeting all CF indicators. Lower household wealth and lower community‐level access to health care services were associated with lower odds of MDD and MAD. Disparities in achieving recommended CF practices were observed by region. CF practices in Afghanistan are poor and significant socioeconomic inequities in CF are observed across the country. Our study calls for urgent policy and programme attention to improve complementary feeding practices as an intrinsic part of the national development agenda.

**Key messages**

- Slightly more than half of Afghan children received complementary foods at 6–8 months of age. Only about half of children aged 6–23 months old were fed with the minimum required frequency; less than a quarter were fed a minimum diverse diet; and less than one fifth were fed a minimum acceptable diet.
- Poor child dietary diversity was driven by low intake of four nutrient‐rich food groups: flesh foods (only 14% of children); fruits and vegetables (15%); legumes and nuts (18%); and eggs (20%).
- Risk factors for poor complementary feeding practices included younger age, poor access to health care services, household poverty, and residence in the Central Highland region.

## 1 INTRODUCTION

Afghanistan has been in near constant conflict, political instability, and economic downturn since the 1970s, (Wieser, Rahimi, & Redaelli, 2017), with a Human Development Index ranking at 169/188 in the world on the basis of indicators of life expectancy, education, and per capita income (United Nations Development Programme, 2016). In 2013 to 2014, about 40% of Afghans lived in poverty, without enough income to cover their basic needs, including food (Wieser et al., 2017). Conflict and economic downturn have put many Afghans at risk of food and nutrition insecurity, especially children aged less than 5 years, who represent about 16% of the country's population (United Nations, 2017) and whose nutrition, growth and development determine the development and prosperity opportunities of the country.

Despite conflict and poverty, under‐five mortality in Afghanistan declined by almost 30% between 2000 and 2015, from 120 to 81 child deaths per 1,000 livebirths (United Nations, 2017). Similarly, stunting among children aged 0–59 months declined by 30%, from 59% in 2004 (World Bank, 2017) to 41% in 2013 (Afghanistan Ministry of Public Health & UNICEF, 2013). However, despite this decline, the prevalence of child stunting remains unacceptably high, as two in five children under five have stunted growth and 1 in 5 are severely stunted (Higgins‐Steele et al., 2016). In addition, the prevalence of child wasting has stagnated at around 10% since 2004 (Afghanistan Ministry of Public Health & UNICEF, 2013; Central Statistics Organisation (CSO), & UNICEF, 2012; World Bank, 2017).

Suboptimal feeding contributes to infant and child mortality (Jones et al., 2003). Its effect is mediated by poor nutritional status (Black et al., 2013; Stewart, Iannotti, Dewey, Michaelsen, & Onyango, 2013) and/or infectious diseases, such as pneumonia, diarrhoea, and others, which are the leading causes of under‐five mortality in Afghanistan (Akseer et al., 2016). The latest data indicate that only 43% of Afghan infants aged 0–5 months are exclusively breastfed (CSO, Ministry of Public Health, & ICF International, 2017). Complementary feeding practices are even poorer, as only 16% of children aged 6–23 months are fed diets that meet the minimum adequacy in terms of feeding frequency and diet diversity (CSO et al., 2017).

Between 2006 and 2015, Afghanistan's Ministry of Public Health adopted a number of policies and strategies to improve maternal and child health and nutrition, with a strong focus on rebuilding urgent health care and services. Though infant and young child nutrition was included in the national child health policy 2004 to 2006 and the national child and adolescent health policy and strategy 2009 to 2013, nutrition has not been sufficiently prioritized in recent national development agendas (Institute of Development Studies, Irish Aid,, & UK Aid, 2014)**.** Supporting a call for greater attention to and investment in nutrition (Varkey, Higgins‐Steele, Mashal, Hamid, & Bhutta, 2015), our study aims to discern the immediate and underlying predictors of complementary feeding of children aged 6–23 months in Afghanistan. We analysed data collected by the 2015 Afghanistan Demographic and Health Survey (AfDHS; CSO et al., 2017), which provided up to date information on child feeding practices from a large nationally representative sample of infants and young children aged 0–23 months. Further, our study aims to identify priority areas of focus for future policies, strategies programs aiming to improve the quality of complementary foods and feeding practices among Afghan children.

## 2 METHODS

### 2.1 Data source

The 2015 AfDHS employed a two‐stage sampling design: At the first stage, a total of 950 clusters (260 urban and 690 rural) were selected from 25,974 census enumeration areas. Given the existence of inaccessible clusters in each of the 34 provinces due to insecurity, 101 reserve clusters were preselected to replace inaccessible clusters, resulting in a total of 1,051 clusters selected at the first stage. At the second stage, 27 households were selected in each cluster through an equal probability systematic sampling process. Excluding clusters that were identified as insecure (*n* = 75), the AfDHS was carried out in 976 of 1,051 clusters and data collection took place from June 15, 2015, to February 23, 2016. The AfDHS could not provide estimates for Zabul Province, where data were collected in only seven urban clusters due to fieldwork challenges.

The data analysed in this study includes information from (a) the household questionnaire, which collected demographic and socioeconomic information; and (b) the women's questionnaire, which collected information from ever‐married women aged 15–49 years, including infant and young child feeding (IYCF) practices in children born in the previous 3 years and living with the respondent at the time of the survey. The response rates to the household questionnaire and the women's questionnaire were 97.8% and 96.8%, respectively.

For the purpose of our analytic sample, we defined eligible children as the youngest singleton child aged 6–23 months old to avoid potential recall bias, and to avoid including children from the same households. Given these inclusion criteria, our descriptive results are slightly different from what has been reported in the 2015 AfDHS final report (CSO et al., 2017).

### 2.2 Complementary feeding indicators

The World Health Organization defines four CF indicators at the population level: introduction of solid, semi‐solid, or soft foods (INTRO), minimum meal frequency (MMF), minimum dietary diversity (MDD), and minimum acceptable diet (MAD; World Health Organization, 2010). The definitions of these CF indicators are summarized in Table 1 and are described briefly below as follows:

**Table 1:** Definition of complementary feeding indicators according to World Health Organization^a^ (World Health Organization, 2010)

	Age group	
	6–8 months	9–23 months
Minimum dietary diversity (MDD)	No. of food groups^b^ ≥ 4	
Minimum meal frequency (MMF)		
Breastfed	No. of solid, semi‐solid, or soft foods ≥2	No. of solid, semi‐solid, or soft foods ≥3
Nonbreastfed	Total # of solid, semi‐solid, or soft foods AND milk feeds^c^ ≥ 4	
Minimum acceptable diet (MAD)		
Breastfed	No. of food groups^b^ ≥ 4, AND	No. of food groups^b^ ≥ 4, AND
	No. of solid, semi‐solid, or soft foods ≥ 2	No. of solid, semi‐solid, or soft foods ≥ 3
Nonbreastfed	No. of food groups^d^ ≥ 4, AND	
	No. of milk feeds^c^ ≥ 2, AND	
	Total no. of solid, semi‐solid, or soft foods AND milk feeds^c^ ≥ 4	

a The complementary feeding indictor would be coded as one if the listed criteria were met by a child per the child's age and feeding mode using child's dietary information in the previous day or night. The table is adapted from (Na et al., 2015)

b Food group score is calculated based on consumption of seven food groups, grains, roots and tubers, legumes and nuts, dairy products, flesh foods, eggs, vitamin A‐rich fruits and vegetables, and other fruits and vegetables.

c Milk feeds are consumption of infant formula, milk such as tinned, powdered or fresh animal milk, and yogurt.

d Food group score is calculated based on consumption of six food groups, excluding dairy products.

*INTRO*: The proportion of infants 6–8 months of age who received solid, semi‐solid, or soft foods in the previous 24 hr (day or night).

*MMF*: The proportion of children aged 6–23 months who received solid, semi‐solid, or soft foods the minimum number of times or more in the previous 24 hr (day or night). For still breastfed children, the MMF is defined as two times for infants aged 6–8 months or three times for children aged 9–23 months. For nonbreastfed children aged 6–23 months, the MMF is four times, including milk feeds.

*MDD*: The proportion of children aged 6–23 months who received foods from four or more food groups in the previous 24 hr (day or night). Seven standard food groups were defined: (a) grains, roots, and tubers; (b) legumes and nuts; (c) dairy products; (d) flesh foods; (e) eggs; (f) vitamin A‐rich fruits and vegetables; and (g) other fruits and vegetables. In addition to MDD, in our study, we also examined the consumption of individual food groups, and we constructed a child dietary diversity score by summing the total number of food groups consumed in the previous 24 hr (day or night); this variable could range from zero (no food group eaten) to seven (all food groups eaten).

*MAD*: The proportion of children aged 6–23 months who received the MDD and the MMF in the previous 24 hr (day or night). For breastfed children, MAD is achieved if the child meets both the MMF and MDD criteria. For nonbreastfed children, the child is required to receive at least four food groups excluding dairy products, two milk feeds, and MMF.

Because there were missing data for milk feed frequency among nonbreastfed children (*n* = 1,509 out of 1,571 nonbreastfed eligible children), MMF and MAD were calculated only for breastfed children. Given that the majority (94%) of children aged 6–8 months were still breastfed, the INTRO indicator largely represents breastfed children. For the ease and consistency of results reporting, we have limited our predictor analysis to breastfed children only for all CF indicators.

### 2.3 Predictor variables

In line with our previous work (Na, Aguayo, Arimond, Narayan, & Stewart, 2018; Na, Aguayo, Arimond, Dahal et al., 2018), we selected the potential predictors of CF practices according to established conceptual frameworks (Black et al., 2013; Stewart et al., 2013), grouping them into two levels: (a) *Individual/household level* and (b) *community level*.

We first selected characteristics of the child, the parents, and the household that represented proximal factors that can potentially predict complementary feeding practices. For children, we included the following variables: sex, age, birth order, birth interval, perceived birth weight, recent vitamin A and iron supplementation status, vaccination record, and recent symptoms of diarrhoea, fever and cough. For mothers, we included the following variables: age, smoking status, and use of reproductive health services: delivery at health facility, delivery with skilled birth assistance, caesarean delivery, number of antenatal care visits, timing of postnatal check‐up for mother and for child, education level, occupation, exposure to media (newspaper, radio, and TV), and empowerment status (involvement in decision‐making for large household purchases, freedom to visit family and friends, own health care, and attitude towards domestic violence regarding five scenarios). Using available data, we calculated a composite women's empowerment score for each mother using an established methodology (Jennings et al., 2014; Na, Jennings, Talegawkar, & Ahmed, 2015). For fathers, we included age, education level, and occupation. Household characteristics included in the analysis were: Sex of household head, household size, number of children under five, type of cooking fuel, water source, time to get to water source, toilet conditions and sharing, and household wealth quintile, which were composed by AfDHS according to a standard principal components analysis method (Rutstein & Johnson, 2004).

Using information from all survey participants, we also included a second‐level of potential predictors at the community level that represented contextual factors influencing child feeding and care. The unit of community in this analysis was the cluster. These factors included place of residence (rural or urban), geographical region, prevalence of women who completed primary or higher education, average women's empowerment score, and prevalence of unimproved toilets and shared toilets. In addition, using a previously developed scoring scheme (Na, Aguayo, Arimond, & Stewart, 2017), we created a composite indicator to rank general community‐level access to health care based on 10 indicators related to child vaccination, reproductive health care services, and coverage of maternal and child supplementation using data from all surveyed subjects (children 0–5 years; women 15–49 years) The rank score characterizing community‐level access to health care is used in the later analysis.

### 2.4 Statistical analysis

Sample characteristics, distribution of complementary feeding practices, dietary diversity score, and individual food group intake were adjusted for the multistage stratified sampling design. Confidence intervals around prevalence and mean values were generated using the Taylor series linearization methods (Wolter, 2007). The linear trend in the prevalence by child age was tested by a score test for trend of odds.

To confirm the number of levels included in the regression analysis, we first constructed intercept only models (null models) for INTRO, MMF, MDD, and MAD, separately, fitting the study sample under one‐level (assuming uncorrelated individuals only) and two‐level (individuals nested within communities) model structures. We then compared the variance explained in the two nested models by the log likelihood ratio (LLR) test. The results of LLR tests indicated that the two‐level models were superior to one‐level models for all four CF indicators (all *P* values <0.0001).

The univariate association for each predictor variable and CF indicator pair was tested using univariate two‐level logistic regression models. The reference group of a categorical predictor variable was generally chosen as the group with the largest weighted sample size. The Wald test was used to test statistical significance of univariate associations under the asymptotic normal distribution assumption.

To investigate independent associations between the predictor variables and CF indicators, we constructed multivariable two‐level logistic regression models for INTRO, MMF, MDD, and MAD, respectively. First, predictor variables that yielded a *P* value less than 0.10 in univariable models were included in the full model. Because 57% and 76% of the respondents had missing data for timing of postnatal check‐ups on women and on children, respectively, the models exclude these two indicators. Type of assistance at delivery was removed because of implausible relationships with the outcome variables in the univariable models, which may indicate errors in data collection of this variable. Second, to avoid collinearity, predictor variables with variance inflation factors (VIFs) greater than five were removed in the descending order of their VIF values. Third, the final set of multivariable models were run with the remaining predictor variables. The multilevel logistic models were developed without adjusting for the multistage sampling design and are considered as internally valid (Rutstein & Rojas, 2006). Fourth, a set of sensitivity analyses were performed: (a) to fix child sex, maternal age, and paternal age, household wealth as covariates in the final models; (b) to include nonbreastfed children in the analysis of MDD (Supplemental Table 3); (c) to include type of assistance at delivery in the model; and (d) to rerun the analysis using multilevel bootstrapping and loosening the estimation assumption of the asymptotic normal distribution.

We used STATA/SE 15.0 (StataCorp, College Station, TX) to analyse data and to generate graphs and tables.

## 3 RESULTS

### 3.1 Sample characteristics

The analytical unweighted sample included 7,963 children from 953 clusters. Demographic and socioeconomic characteristics by individual, household, and community levels are presented in Table 2. After weighting on survey design, about 80% of children were being breastfed at the time of the survey. About half of the children (47%) were second to fourth births. More than half (55%) were born within a 24‐month birth interval. More than half (61%) were perceived as having an “average” weight at birth. Less than half (45%) had completed all the vaccinations scheduled for their age. About two‐thirds (69%) of mothers were between 25 and 34 years. Although 50% of the mothers delivered their children with the assistance of a nurse or a doctor, less than 20% of mothers benefitted from four or more antenatal check‐ups. About 80% of mothers did not have any formal education and most of them (87%) did not work outside their homes. The prevalence of fathers without formal education was 56%, all of the fathers were working, and most of them (73%) worked in nonagricultural sectors. Households comprised on average 10 people with about three children under 5 years of age. The majority (96%) of households had access to water within 1 hr walking distance but in one‐third (32%) of households drinking water was provided by unimproved sources. Two‐thirds (67%) of the households did not have improved toilet facilities. Most eligible communities (73%) were rural. Across communities, the median prevalence of community‐level access to health care ranged from 0% for child iron supplementation in the last 7 days to 56% for health facility delivery.

**Table 2:** Demographic and socioeconomic characteristics at individual, household, and community level in Afghanistan 2015

	*N*	Percent or mean (*SE*)
Child characteristics		
Female	7,936	48.6
Still breastfed	7,930	79.6
Age (months)	7,936	
6–11		33.7
12–17		44.6
18–23		21.6
Birth order	7,936	
Firstborn		18.3
Second to fourth		46.8
Fifth and more		34.9
Birth interval (month)	7,936	
No previous birth		18.3
<24		26.3
> = 24		55.4
Perceived birth weight	7,765	
Smaller than average		24.3
Average		60.9
Larger than average		14.8
Received vitamin A supplementation in the past 6 months	7,562	49.6
Received iron pills, sprinkles or syrup in the last 7 days	7,595	6.1
Complete age‐appropriate vaccination	7,692	43.5
Child health: Had the following symptom in the past 2 weeks		
Diarrhoea	7,866	37.4
Fever	7,885	36.1
Cough	7,859	27.8
Maternal characteristics		
Age (years)	7936	27.5 (0.12)
15–24		5.7
25–34		68.5
35–49		25.8
Smoker	7,912	3.6
Reproductive health care		
Delivered at health facility	7,929	53.5
Type of delivery assistance	7,931	
Health professional		49.6
Traditional birth attendant		30.0
Other		20.4
Caesarean delivery	7,935	3.7
Antenatal clinic visits	7,786	
None		37.4
1–3	,	43.3
≥4		19.3
Postnatal check‐up on woman	7,936	
0‐1d		37.4
> = 2d		6.1
Missing or unknown		56.5
Postnatal check‐up on child	7,936	
0‐1d		7.3
> = 2d		17.0
Missing or unknown		75.7
Highest educational level	7,936	
No education		80.1
Primary		8.8
Secondary or higher		11.0
Occupation	7,928	
Not working		87.4
Agricultural		1.6
Non‐agricultural		11.0
Currently married	7,936	99.4
Exposure to media: At least once a week		
Reading newspaper	7,917	2.7
Listening to radio	7,932	24.2
Watching TV	7,916	38.7
Involved in decision making on		
How man's income is used	7,834	30.8
Large household purchases	7,857	41.2
Visiting family and friends	7,858	51.6
Regarding own health care	7,859	47.0
Attitude towards domestic violence: Beating justified if		
Goes out without telling him	7,896	68.7
Neglects the children	7,899	50.4
Argues with him 7899		60.3
Refuses to have sex with him 7894		33.5
Burns the food	7,900	19.2
None above	7,901	14.1
Women's empowerment score (five items)	7,936	1.8 (0.05)
Paternal characteristics		
Age (years)	7,852	32.5 (0.16)
15–24		51.0
25–34		13.0
> = 35		36.1
Highest educational level	7,853	
No education		56.1
Primary		14.5
Secondary or higher		29.3
Occupation	7,866	
Agricultural		27.5
Nonagricultural		72.5
Household characteristics		
Female household head	7,936	1.1
No. of household members	7,852	9.7 (0.20)
No. of children under 5 years	7,936	2.6 (0.05)
Types of cooking fuel	7,888	
Electricity, LPG, natural gas, biogas		32.4
Wood, straw/shrubs/grass, animal dung and other		67.6
Water source		
Unimproved source of drinking water	7,933	32.4
Source for water not in own dwelling or yard/plot	7,324	58.2
Time to get to water source (min)	7,835	
0		46.5
1–59		49.4
> = 60		4.1
Toilet condition		
Unimproved toilet facility	7,932	66.6
Shared toilet with other households	7,894	28.7
HH wealth	7,936	
Poorest		18.0
Poorer		19.3
Middle		20.7
Richer		22.1
Richest		19.9
Community characteristics	*N* of clusters	% or mean (*SD*) or medium [IQR]
Rural residence	953	72.8
Geographical region	953	
Northern		14.7
North Eastern		12.0
Western		12.9
Central Highland		5.9
Capital		18.6
Southern		15.2
South Eastern		8.9
Eastern		11.9
% women completed primary or higher education	953	16.1 (16.0)
Mean women's empowerment	953	2.0 (1.0)
Access to health care		
% children completed age‐appropriate vaccine	953	37.5 [20.0, 54.5]
% delivered at health facility	953	56.3 [26.1, 77.3]
% delivered with professional assistance	953	50.0 [15.2, 73.7]
% had caesarean delivery	953	0.0 [0.0, 5.0]
% had > = 4 ANC visits	953	13.0 [0.0, 29.2]
% postnatal check‐up on woman within 1 day of delivery	953	30.8 [12.0, 52.9]
% postnatal check‐up on child within 1 day of delivery	953	4.0 [0.0, 10.5]
% children 0‐5y received vitamin A in the last 6 months	953	45.0 [23.3, 65.7]
% children 0‐5y received iron pills, sprinkles or syrup in the last 7 days	953	0.0 [0.0, 9.1]
% women given or bought iron tablets during pregnancy	953	40.0 [19.4, 59.1]
Average rank of access to health care	953	424.1 (149.6)
Sanitation condition		
% unimproved toilet	953	83.3 [53.6, 100.0]
% sharing toilet with other households	953	22.5 [10.3, 39.4]

*Note*. IQR: interquartile range; *SE*: standard error; *SD*: standard deviation.

### 3.2 Distribution of CF practices and individual food group intake

**Figure 1:**
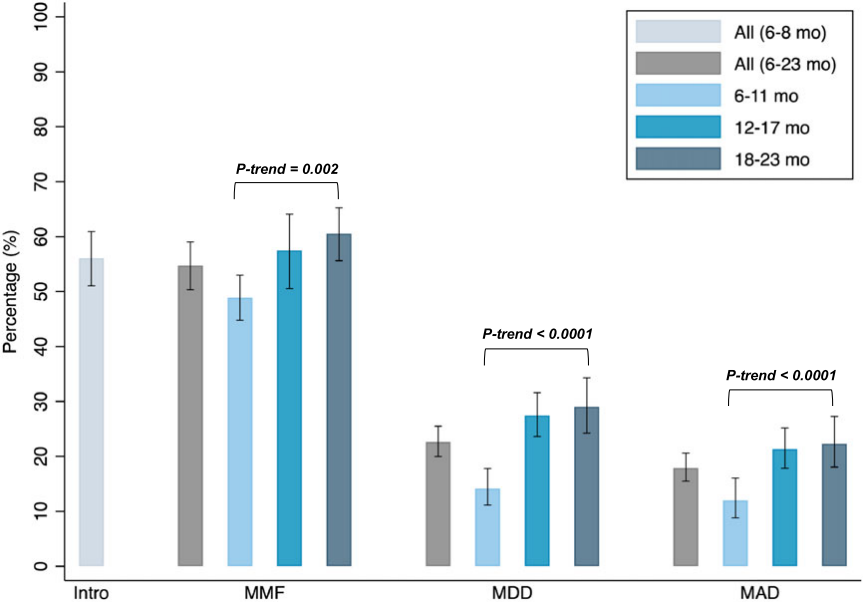
Prevalence of children meeting the WHO defined complementary feeding indicators in Afghanistan in 2015. Only breastfed children are included. Error bars are the lower and upper 95% confidence bounds of the prevalence. INTRO, introduction of solid, semi‐solid, and soft food; MMF, minimum meal frequency; MDD, minimum dietary diversity; MAD, minimum acceptable diet

The weighted sample sizes for INTRO, MMF, MDD, and MAD were 1,323, 5,486, 6,151, and 5,606, respectively. The prevalence 95% confidence interval (CI) of respondents reporting recommended CF practices is presented for all eligible breastfed children and by age (Figure 1). The prevalence of INTRO among all children aged 6–8 months was 56% (51% and 61%). The prevalence of MMF, MDD, and MAD among breastfed children aged 6–23 months (i.e., 80% of the total sample) was 55% (50%, 59%), 23% (20%, 25%) and 18% (15%, 21%), respectively. There was a significant increasing trend of MMF, MDD, and MAD with increasing child age (*P* trend values were 0.002, <0.001, and <0.001, respectively).

**Figure 2:**
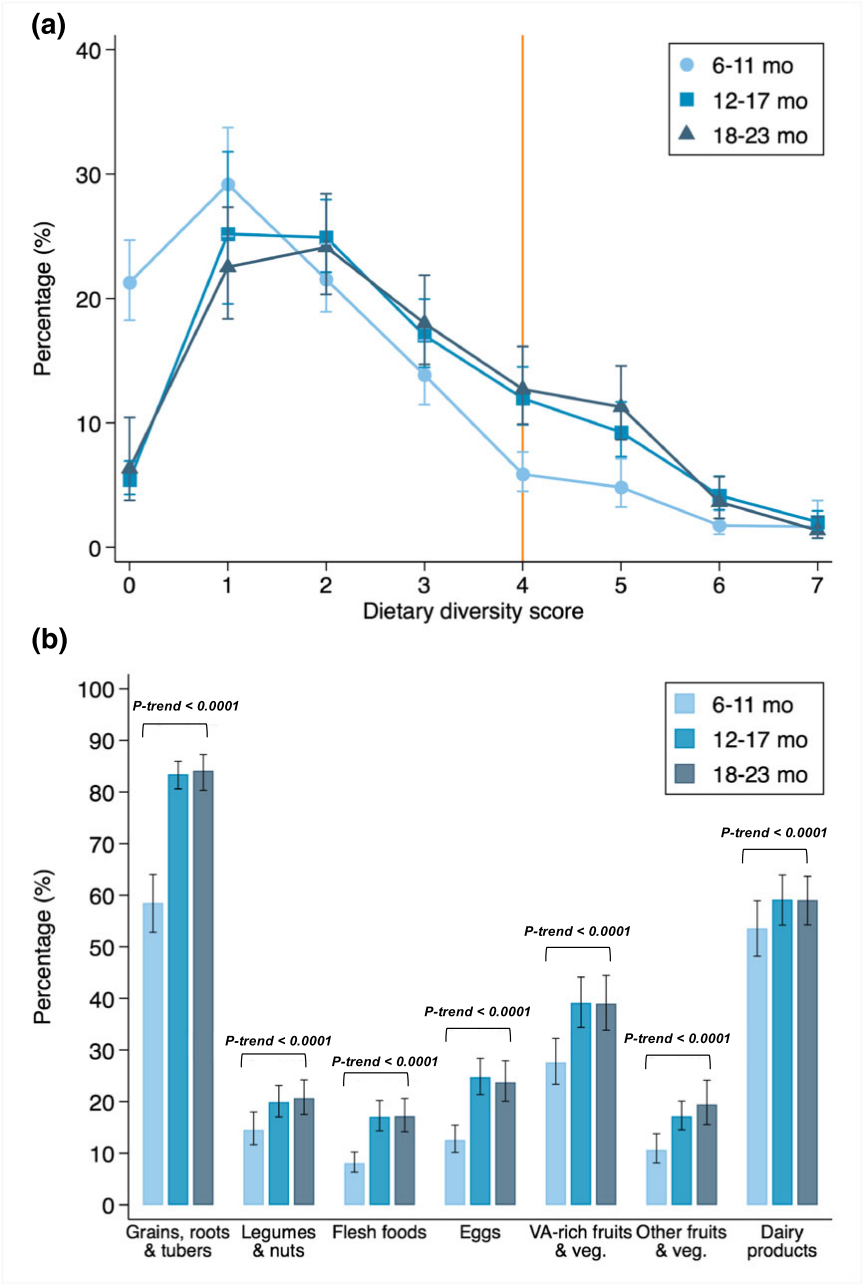
The distribution of a dietary diversity score (a) and the prevalence of consuming individual food groups (b) among breastfed children in Afghanistan. Only breastfed children are included. The orange vertical line indicates the minimum required four food groups per WHO's recommendation. Error bars are the lower and upper 95% confidence bounds of the prevalence. *P* trend value for dietary diversity by age is less than 0.001 predictors of complementary feeding practices in Afghanistan. An in‐depth analysis of the 2015 Demographic and Health Survey

The weighted mean dietary diversity score (95% CI) was 2.4 (2.3, 2.6) in all children, and 1.9 (1.8, 2.0), 2.7 (2.6, 2.9), and 2.7 (2.6, 2.9) among children aged 6–11, 12–17, and 18–23 months, respectively (Figure 2a). Similarly, there was a significant trend of increasing dietary diversity with child age (*P* trend <0.001). Corresponding to the overall trend, the prevalence of children consuming individual food groups (Figure 2b) also increased significantly with child age (all *P* trend <0.0001). The most evident increase in intake occurred between children 6–11 months and 12–17 months, where there was a 25% point increase for grains, roots, and tubers (from 58% to 83%), a 12% point increase for eggs (from 13% to 25%), a 12% point increase for vitamin‐A rich fruits and vegetables (from 28% to 39%), and a 9% point increase for flesh foods (from 8% to 17%). The magnitude of the increase by child age for the other three food groups was smaller, likely because, the prevalence of children eating legumes and nuts, and other fruits and vegetables was universally low regardless of child age (<20%), whereas the prevalence of eating dairy products was universally high (>50%). Despite significant improvement with age, less than 20% of all children—regardless of age—consumed four or more food groups. Consumption was particularly low in the case of flesh foods, at 14% (12%, 16%), other fruits and vegetables, at 15% (13%, 17%), legumes and nuts, at 18% (16%, 20%) and eggs, at 20% (18%, 22%). Vitamin A‐rich fruits and vegetables and dairy products were consumed by 35% (31%, 38%) and 57% (54%, 59%) of all children. Grains, roots and tubers was the most commonly consumed food group, reported by 74% (72%, 76%).

### 3.3 Predictors of CF indicators

The adjusted odds ratio (OR) and 95% CI for predictors of CF indicators in the multivariable multilevel models are presented in Table 3. Comparing children 12–17 months and 18–23 months to children aged 6–11 months (reference group), the adjusted odds of meeting the MMF increased by 2.2–2.3 fold, meeting the MDD increased by 3.4–3.9 fold, and meeting the MAD increased by 3.1–3.4 fold. Compared with the second‐to‐fourth born children, firstborn children had ~20% lower odds of MMF and MAD, whereas children who were born as fifth or later child in the family had ~20% lower odds of MAD. Children who were perceived to be greater than average at birth had a ~50% and ~30% increased odds of meeting INTRO and MDD, respectively, than children whose birth weight was perceived to be “average” (reference). Children who had none of their vaccinations had ~60% lower odds of INTRO than children who had all the age‐appropriate vaccinations, controlling for all other covariates at individual, household, and community level.

**Table 3:** Predictors of meeting complementary feeding practice using multivariable multilevel logistic regression analysis among breastfed children in Afghanistan

	Intro			MMF			MDD			MAD		
	Estimate		*P* value	Estimate		*P* value	Estimate		*P* value	Estimate		*P* value
	OR	(95% CI)		OR	(95% CI)		OR	(95% CI)		OR	(95% CI)	
*N*		1,234			5,394			5,905			5,178	
Child characteristics												
Age (months)												
6–11				1.00	(referent)		1.00	(referent)		1.00	(referent)	
12–17				2.25	(1.93, 2.61)	***	3.42	(2.80, 4.18)	***	3.10	(2.48, 3.89)	***
18–23				2.32	(1.91, 2.81)	***	3.90	(3.06, 4.96)	***	3.37	(2.56, 4.43)	***
Birth order												
Firstborn	1.20	(0.84, 1.72)	0.32	0.81	(0.67, 0.99)	*	0.85	(0.66, 1.08)	0.18	0.76	(0.58, 0.99)	*
Second to fourth	1.00	(referent)		1.00	(referent)		1.00	(referent)		1.00	(referent)	
Fifth and more	1.34	(0.99, 1.81)	0.06	0.93	(0.80, 1.08)	0.36	0.92	(0.74, 1.15)	0.45	0.81	(0.66, 1.00)	*
Perceived birth weight												
Smaller than average	1.32	(0.87, 2.02)	0.19				1.22	(0.99, 1.50)	0.06			
Average	1.00	(referent)					1.00	(referent)				
Larger than average	1.55	(1.12, 2.15)	**				1.27	(1.01, 1.60)	*			
Age‐appropriate vaccination												
None	0.39	(0.25, 0.61)	***							1.21	(0.88, 1.66)	0.25
Some	0.90	(0.66, 1.23)	0.51							0.94	(0.76, 1.17)	0.58
Complete	1.00	(referent)								1.00	(referent)	
Maternal characteristics												
Antenatal clinic visits												
None	1.00	(referent)		1.00	(referent)		1.00	(referent)		1.00	(referent)	
1–3	1.63	(1.16, 2.27)	**	1.30	(1.11, 1.53)	**	1.55	(1.27, 1.90)	***	1.51	(1.20, 1.91)	***
≥4	1.34	(0.87, 2.05)	0.18	1.43	(1.15, 1.77)	**	1.42	(1.09, 1.85)	**	1.45	(1.07, 1.96)	*
Education												
No education	1.00	(referent)		1.00	(referent)		1.00	(referent)		1.00	(referent)	
Primary	1.62	(0.98, 2.67)	0.06	1.35	(1.04, 1.75)	*	1.13	(0.83, 1.55)	0.43	1.16	(0.82, 1.64)	0.39
Secondary or higher	1.10	(0.67, 1.80)	0.71	1.02	(0.79, 1.33)	0.86	1.45	(1.06, 1.98)	*	1.34	(0.95, 1.89)	0.09
Occupation												
Not working							1.00	(referent)		1.00	(referent)	
Agricultural							0.48	(0.25, 0.90)	*	0.51	(0.25, 1.06)	0.07
Non‐agricultural							0.72	(0.52, 1.01)	0.06	0.83	(0.57, 1.21)	0.34
Paternal characteristics												
Education												
No education	1.00	(referent)		1.00	(referent)		1.00	(referent)				
Primary	0.67	(0.45, 1.01)	0.06	0.84	(0.69, 1.03)	0.09	0.98	(0.76, 1.26)	0.85			
Secondary or higher	0.82	(0.58, 1.15)	0.25	0.84	(0.71, 1.00)	*	1.04	(0.85, 1.29)	0.69			
Household characteristics												
Time to get to water source (min)												
0				1.00	(referent)							
1–59				1.00	(0.84, 1.18)	0.96						
> = 60				0.70	(0.48, 1.03)	0.07						
HH wealth												
Richest	1.00	(referent)					1.00	(referent)		1.00	(referent)	
Richer	1.52	(0.96, 2.42)	0.07				0.50	(0.37, 0.68)	***	0.61	(0.43, 0.86)	**
Middle	1.28	(0.80, 2.05)	0.30				0.56	(0.40, 0.78)	**	0.65	(0.45, 0.95)	*
Poorer	2.08	(1.26, 3.42)	**				0.76	(0.54, 1.06)	0.11	0.84	(0.57, 1.24)	0.39
Poorest	2.07	(1.20, 3.56)	**				0.68	(0.47, 1.01)	0.05	0.78	(0.51, 1.21)	0.27
Community characteristics												
Geographical region												
Northern	1.00	(referent)		1.00	(referent)		1.00	(referent)		1.00	(referent)	
North Eastern	0.46	(0.26, 0.79)	**	1.56	(1.04, 2.34)	*	0.98	(0.61, 1.57)	0.92	0.95	(0.57, 1.57)	0.83
Western	2.22	(1.26, 3.88)	**	1.86	(1.25, 2.78)	**	0.80	(0.50, 1.29)	0.37	0.75	(0.46, 1.24)	0.27
Central Highland	0.27	(0.13, 0.53)	***	0.70	(0.42, 1.18)	0.18	0.28	(0.14, 0.55)	***	0.07	(0.03, 0.21)	*
Capital	0.58	(0.34, 1.00)	*	1.04	(0.71, 1.53)	0.83	0.87	(0.56, 1.36)	0.55	0.81	(0.50, 1.32)	0.40
Southern	1.24	(0.69, 2.23)	0.46	1.41	(0.94, 2.12)	0.10	0.55	(0.33, 0.90)	*	0.37	(0.21, 0.66)	**
South Eastern	0.50	(0.26, 0.97)	*	0.80	(0.52, 1.24)	0.33	0.94	(0.55, 1.62)	0.83	1.05	(0.60, 1.85)	0.86
Eastern	0.97	(0.57, 1.66)	0.92	1.54	(1.03, 2.30)	*	0.97	(0.60, 1.58)	0.91	1.03	(0.61, 1.74)	0.92
Rank of access to health care												
Highest (best access)	1.00	(referent)					1.00	(referent)		1.00	(referent)	
Higher	0.66	(0.41, 1.05)	0.08				0.59	(0.39, 0.88)	*	0.56	(0.36, 0.87)	**
Medium	0.63	(0.38, 1.06)	0.08				0.46	(0.30, 0.71)	***	0.46	(0.29, 0.74)	**
Lower	0.47	(0.27, 0.82)	**				0.71	(0.45, 1.12)	0.14	0.57	(0.35, 0.94)	*
Lowest (worse access)	0.83	(0.47, 1.47)	0.51				0.61	(0.38, 0.98)	*	0.37	(0.21, 0.63)	***

*Note*. Intro = introduction of solid, semi‐solid, and soft foods; MMF = minimum meal frequency; MDD = minimum dietary diversity; MAD = minimum acceptable diet; OR = odds ratio; CI = confidence interval.

* *P* < 0.05.

** *P* < 0.01.

*** *P* < 0.001.

Children of mothers who attended antenatal clinic visits more than once had 1.3–1.6 times higher odds of meeting INTRO, MMF, MDD, and MAD than children whose mothers did not attend antenatal clinic visits. Maternal education was not significantly associated with INTRO or MAD; however maternal primary education was associated with a greater odds of meeting MMF and maternal secondary education or higher was associated with a greater odds of meeting MDD compared with children of mothers without formal education (reference group).

Using the wealthiest quintile as the reference, the odds of meeting INTRO were ~2 fold higher among children of the two bottom wealth quintiles. However, compared with children from the wealthiest quintile, children from the richer and middle wealth quintiles had a ~40% lower odds of meeting MDD and MAD criteria. The odds of meeting CF indicators varied substantially by region. Compared with the Northern region, the Central Highland region had the lowest relative odds for INTRO (OR = 0.27, 95% CI [0.13, 0.53]), MDD (OR = 0.28, 95% CI [0.14, 0.55]), and MAD (OR = 0.07, 95% CI [0.03, 0.21]), the Western region had the highest relative odds for INTRO (OR = 2.22, 95% CI [1.26, 3.88]), and MMF (OR = 1.86, 95% CI [1.25, 2.78]). Communities in quintiles of lower rank in access to health care, had lower odds of meeting INTRO, MDD, and MAD than communities in quintiles with highest rank in access to health.

Our sensitivity analyses revealed similar OR in models with child sex, maternal age, paternal age, and household wealth fixed as covariates, with nonbreastfed children included in the analysis of MDD, with type of delivery assistance variable included, and with bootstrapping analysis adjusting for potential bias of nonnormality of the statistics being estimated (data not shown).

## 4 DISCUSSION

Using nationally representative data from Afghanistan's 2015 DHS, we present updated estimates on CF practices and the factors associated with them among breastfed children aged 6–23 months living with their mothers. Only slightly more than half (56%) of children aged 6–8 months were fed solid, semi‐solid, or soft foods and only slightly more than half (55%) of children aged 6–23 months were fed the minimum recommended number of times per day. Child MDD and MAD were both extremely poor as less than one‐fifth of children aged 6–23 months achieved these criteria. Current complementary feeding practices in Afghanistan are among the poorest in South Asia, a region where only 15–32% of children meet the MAD criteria (International Institute for Population Sciences, M. I, 2007; Na, Aguayo, Arimond, Dahal, et al., 2018; Na, Aguayo, Arimond, & Stewart, 2017; National Institute of Population Research and Training, Mitra and Associates,, & ICF International, 2016).

Between 2003 and 2015, the prevalence of children being introduced to complementary foods in a timely manner showed significant improvement, from 28.9% in 2003 (Central Office of Statistics & UNICEF, 2004), to 56% in 2015 according to our analysis. The prevalence of children achieving MMF increased significantly between 2010 (18%) and 2013 (52%), showing no improvement thereafter. MDD among children aged 6–23 months declined from 28% in 2013 to 23% in 2015. As a result, the prevalence of children achieving MAD, which includes both meal frequency and diet diversity remained at a low 14–18% between 2013 and 2015. Challenges with field data collection and/or small sample sizes in these surveys lend uncertainty to these estimates. However, the slight decline in child dietary diversity since 2013 indicates the lack of impact of current policies, strategies, and programmes to improve the diversity and overall adequacy of children's diets.

Poor dietary diversity during complementary feeding puts fast‐growing infants and young children at risk of inadequate intakes for essential micronutrients, with the greatest gaps usually found for iron and zinc (Dewey, 2013). Poor dietary diversity in children has been associated with low height‐for‐age *z* scores and stunting in many low‐ and middle‐income countries (Arimond & Ruel, 2004; Mallard et al., 2014; Rah et al., 2010). In Afghanistan, inadequate dietary diversity was associated with 34% increased odds of stunting among children 6–23 months, independent of the influence of breastfeeding, feeding frequency, maternal stature, and other risk factors and covariates (Kim, Mejía‐Guevara, Corsi, Aguayo, & Subramanian, 2017).

Our analysis suggests several opportunities for improving complementary feeding outcomes in Afghanistan:

First, household poverty and poor maternal education need to be addressed to improve complementary feeding practices, especially dietary diversity. Living in deprived households as indicated by the household wealth index, was an independent risk factor to poor child dietary diversity and the overall adequacy of children's diets, an observation that has been consistently found in other South Asian settings (Na, Aguayo, Arimond, Dahal, et al., 2018; Na, Aguayo, Arimond, & Stewart, 2017). Economic constraints at the household level make improving children's dietary diversity challenging, as most nutrient‐rich foods that are lacking in the diets of Afghan children are more expensive and less accessible under conditions of poverty and crisis (Bouis, Eozenou, & Rahman, 2011; Dewey, 2016; Ruel, Garrett, Hawkes, & Cohen, 2010). Maternal education was another socioeconomic factor that predicted meal frequency and dietary diversity. Afghan women have been extremely deprived of educational opportunities, as evidenced by the greater than 80% female illiteracy rate and 80% of women reporting no formal education (CSO et al., 2017). This means that the majority of children are cared for by mothers with limited access to information and are at risk of inappropriate feeding and care. It calls for education policies and programmes that promote access to equitable primary and secondary education for girls in Afghanistan.

Second, knowledge, attitudes, and practices need to be aligned with children's nutritional needs, where household poverty is not a constraint to appropriate complementary feeding practices. Both timing of complementary food introduction and meal frequency practices are likely less constrained by household wealth, as repeatedly seen in the literature (Joshi, Agho, Dibley, Senarath, & Tiwari, 2012; Kabir et al., 2012; Na, Aguayo, Arimond, Dahal, et al., 2018; Na, Aguayo, Arimond, Narayan, et al., 2018; Na, Aguayo, Arimond, & Stewart, 2017; Senarath, Godakandage, Jayawickrama, Siriwardena, & Dibley, 2012) and are less likely to be associated with general socioeconomic progress over time (Na, Aguayo, Arimond, Dahal, et al., 2018; Na, Aguayo, Arimond, Narayan, et al., 2018). Our analysis indicates that timely introduction of complementary foods was not constrained by household wealth. Household wealth was not a barrier to introducing children aged 6–8 months to complementary foods, yet many children aged 6–8 months were not fed complementary foods. Afghan families may have inaccurate perceptions about what appropriate foods for young children are. A recent ethnographic study conducted in four ethnically and geographically diverse areas indicated that foods specifically prepared for infants and young children were uncommon. Further, study participants frequently mentioned “family foods for everyone” as the guide for food procurement and consumption, without routine use of special, home‐prepared complementary foods for young children (Pelto, Armar‐Klemesu, Siekmann, & Schofield, 2013).

Third, the coverage of maternal and child health care at both community and individual level needs to be expanded, and where health care is reaching families, opportunities to improve complementary feeding need to be maximized. As the result of national efforts to rebuild essential health services and programmes, the number of skilled health professionals in Afghanistan increased by 8–15 fold between 2005 and 2013, and the coverage of child vaccination doubled over the same period (Akseer et al., 2016). We found that greater individual‐level and community‐level access to health care was associated with improved complementary feeding practices. However, progress in the availability of health services has not translated into progress on key complementary feeding practices. One possible explanation is that although complementary feeding is included in the basic package of services at policy level, appropriate behaviour‐change counselling for improved complementary feeding practices has not yet been widely implemented as part of facility‐ and community‐based health services. In the recently launched Afghanistan Zero Hunger Strategic Review, a key consultative recommendation to improve child nutrition was to revise the community‐based nutrition intervention package to include counselling on infant and young child feeding (Advisory Committee for Consultations, 2017). There may be other opportunities to further incorporate child feeding modules in the training of health workers' training and performance incentive. Summarizing data from 10 randomized controlled trials, a systematic review concluded that such integration was effective in increasing child energy intake, feeding frequency, and dietary diversity (Sunguya et al., 2013). Another potential channel is to integrate child feeding and nutrition education into vaccination campaigns as an add‐on component, though there is little evidence about compatibility and cost of such integration (A. Wallace, Dietz, & Cairns, 2009; A. S. Wallace, Ryman, & Dietz, 2012). Training health care professionals and integrating nutrition education during vaccination campaigns as part of the information, education, and counselling interventions (Aguayo, 2017) may enhance the educational pathway to address disparities in complementary feeding practices in Afghanistan.

Fourth, vast disparities in IYCF indicators call for prioritized and diversified regional policies to promote complementary feeding practices. Additional analysis revealed that key predictors of feeding practices varied significantly by region (see Supplemental Table 4). Similar to the economic statistics published by World Bank (World Bank, 2016), there is considerably higher proportion of families in the poorest quintile in the Central Highland, Western, and North Eastern regions of the country, where poverty reduction needs to be prioritized if child dietary diversity is to improve. Whereas the Southern region is characterized by indicators of poor maternal education (94% with no education) and worse access to health care and services (52% in the lowest access quintile). Strategies and programmes to improve the quality of complementary must be informed by a good understanding of subnational level constrains to optimal infant and young child feeding and nutrition.

Several limitations of the study must be recognized, including: (a) the cross‐sectional nature of AfDHS, (b) missing data in Zabul Province and other parts of Afghanistan due to insecurity, and (c) the need to limit predictor analysis to breastfed children given missing information for milk feed frequency for non‐breastfed children. Regardless of these limitations, we analysed comprehensively predictors of complementary feeding practices using nationally representative data. The statistical methods took into account the complex survey design and multi‐level structure of the data. Our results remained robust in multiple sensitivity analyses.

In 2015, complementary feeding practices among Afghan children aged 6–23 months were very poor, with virtually no improvement since 2013. Low dietary diversity is primarily attributable to low intake of flesh foods, fruits and vegetables, legumes and nuts, and eggs. Disparities in complementary feeding practices are evident by individual, household and community characteristics. This calls for urgent policy and programme attention to prioritize, integrate, and scale up child nutrition programmes as a key component of the postcrisis national health and development agenda.

## CONFLICTS OF INTEREST

The authors declare that they have no conflicts of interest.

## CONTRIBUTIONS

MN, VMA, MA and CPS conceptualized the research question. MN requested data, conducted literature review, data analysis and prepared the first draft of the manuscript. VMA, MA, PM and CPS provided technical support on study methods, insights on results interpretation and manuscript revisions. All authors read and approved the final manuscript.

## Supporting information


**Table S3**. Univariate and multivariate associations between individual‐household and community level predictors and MDD in non‐breastfed children in multilevel logistic regression analysis.

**Table S4**. Key demographic and socio‐economic predictors of complementary feeding practices by region in Afghanistan 2015.
