# Supplementary material for: Predictors of complementary feeding practices in Afghanistan: Analysis of the 2015 Demographic and Health Survey
Source: Matern Child Nutr. 2018 Nov 29;14(Suppl 4):e12696. doi: 10.1111/mcn.12696 (PMC6587761; doi:10.1111/mcn.12696)
Supplement: Supplementary file 1 — Table S3. Univariate and multivariate associations between individual‐household and community level predictors and MDD in non‐breastfed children in multilevel logistic regression analysis. [file MCN-14-e12696-s001.docx]

| Supplemental Table 3: Univariate and multivariate associations between individual-household and community level predictors and MDD in non-breastfed children in multilevel logistic regression analysis | | | | | | | | |
| --- | --- | --- | --- | --- | --- | --- | --- | --- |
|  |  |  | Univariate association | | | Multivariate association | | |
|  |  |  | Estimate | | *P-value* | Estimate | | *P-value* |
|  |  |  | OR | (95%CI) |  | OR | (95%CI) |  |
| *Child characteristics* | | |  |  |  |  |  |  |
|  | Female | | 1.16 | (0.85, 1.58) | *0.35* |  |  |  |
|  | Age (months) | |  |  |  |  |  |  |
|  |  | 6-11 | 1.00 | (Referent) |  | 1.00 | (Referent) |  |
|  |  | 12-17 | 1.80 | (1.09, 3.00) | *** | 1.61 | (0.95, 2.74) | *0.08* |
|  |  | 18-23 | 1.41 | (0.84, 2.37) | *0.19* | 1.36 | (0.79, 2.33) | *0.27* |
|  | Birth order | |  |  |  |  |  |  |
|  |  | Firstborn | 1.62 | (1.09, 2.41) | *** | 1.48 | (0.97, 2.26) | *0.07* |
|  |  | Second to fourth | 1.00 | (Referent) |  | 1.00 | (Referent) |  |
|  |  | Fifth and more | 1.09 | (0.76, 1.57) | *0.63* | 1.02 | (0.69, 1.51) | *0.92* |
|  | Birth interval (month) | |  |  |  |  |  |  |
|  |  | No previous birth | 1.49 | (1.00, 2.20) | *** |  |  |  |
|  |  | <24 | 0.88 | (0.61, 1.27) | *0.49* |  |  |  |
|  |  | >=24 | 1.00 | (Referent) |  |  |  |  |
|  | Perceived birth weight | |  |  |  |  |  |  |
|  |  | Smaller than average | 1.20 | (0.83, 1.74) | *0.34* |  |  |  |
|  |  | Average | 1.00 | (Referent) |  |  |  |  |
|  |  | Larger than average | 1.39 | (0.90, 2.16) | *0.14* |  |  |  |
|  | Received vitamin A supplementation in the past 6 months | | 2.50 | (1.76, 3.56) | **** |  |  |  |
|  | Received iron pills, sprinkles or syrup in the last 7 days | | 1.35 | (0.64, 2.84) | *0.43* |  |  |  |
|  | Age-appropriate vaccination | |  |  |  |  |  |  |
|  |  | None | 0.36 | (0.21, 0.61) | **** | 0.59 | (0.33, 1.07) | *0.08* |
|  |  | Some | 0.48 | (0.34, 0.69) | **** | 0.59 | (0.40, 0.86) | **** |
|  |  | Complete | 1.00 | (Referent) |  | 1.00 | (Referent) |  |
|  | Child health: had the following symptom in the past 2 weeks | |  |  |  |  |  |  |
|  |  | Diarrhea | 0.89 | (0.64, 1.25) | *0.52* |  |  |  |
|  |  | Fever | 0.75 | (0.53, 1.06) | *0.11* |  |  |  |
|  |  | Cough | 1.02 | (0.69, 1.51) | *0.91* |  |  |  |
| *Maternal characteristics* | | |  |  |  |  |  |  |
|  | Age (years) | |  |  |  |  |  |  |
|  |  | 15-24 | 0.82 | (0.40, 1.68) | *0.59* |  |  |  |
|  |  | 25-34 | 1.00 | (Referent) |  |  |  |  |
|  |  | 35-49 | 1.07 | (0.75, 1.53) | *0.72* |  |  |  |
|  | Smoker | | 0.63 | (0.24, 1.66) | *0.35* |  |  |  |
|  | Reproductive health care | |  |  |  |  |  |  |
|  |  | Delivered at health facility | 1.73 | (1.23, 2.43) | **** |  |  |  |
|  |  | Type of delivery assistance |  |  |  |  |  |  |
|  |  | Health professional | 1.00 | (Referent) |  | 1.00 | (Referent) |  |
|  |  | Traditional birth attendant | 0.53 | (0.34, 0.81) | **** | 1.39 | (0.81, 2.38) | *0.23* |
|  |  | Other | 0.54 | (0.35, 0.82) | **** | 0.87 | (0.53, 1.44) | *0.60* |
|  |  | Caesarean delivery | 1.31 | (0.49, 3.56) | *0.59* |  |  |  |
|  |  | Antenatal clinic visits |  |  |  |  |  |  |
|  |  | None | 1.00 | (Referent) |  | 1.00 | (Referent) |  |
|  |  | 1-3 | 1.85 | (1.27, 2.68) | **** | 1.17 | (0.78, 1.76) | *0.44* |
|  |  | ≥4 | 1.99 | (1.25, 3.17) | **** | 1.09 | (0.64, 1.86) | *0.75* |
|  |  | Postnatal check-up on woman |  |  |  |  |  |  |
|  |  | 0-1d | 2.04 | (1.41, 2.94) | **** |  |  |  |
|  |  | >=2d | 1.73 | (0.90, 3.30) | *0.10* |  |  |  |
|  |  | Missing or unknown | 1.00 | (Referent) |  |  |  |  |
|  |  | Postnatal check-up on child |  |  |  |  |  |  |
|  |  | 0-1d | 0.94 | (0.49, 1.79) | *0.84* |  |  |  |
|  |  | >=2d | 1.33 | (0.85, 2.09) | *0.22* |  |  |  |
|  |  | Missing or unknown | 1.00 | (Referent) |  |  |  |  |
|  | | | | | | | | |
| *Supplemental Table 3 cont’* | | | | | | | | |
|  |  |  | Univariate association | | | Multivariate association | | |
|  |  |  | Estimate | | *P-value* | Estimate | | *P-value* |
|  |  |  | OR | (95%CI) |  | OR | (95%CI) |  |
|  | Maternal education | |  |  |  |  |  |  |
|  |  | No education | 1.00 | (Referent) |  | 1.00 | (Referent) |  |
|  |  | Primary | 1.86 | (1.03, 3.33) | *** | 1.58 | (0.84, 2.97) | *0.16* |
|  |  | Secondary or higher | 2.05 | (1.21, 3.49) | **** | 1.33 | (0.72, 2.46) | *0.36* |
|  | Occupation | |  |  |  |  |  |  |
|  |  | Not working | 1.00 | (Referent) |  | 1.00 | (Referent) |  |
|  |  | Agricultural | 0.42 | (0.16, 1.13) | *0.08* | 0.78 | (0.25, 2.39) | *0.66* |
|  |  | Non-agricultural | 0.63 | (0.32, 1.22) | *0.17* | 0.50 | (0.24, 1.06) | *0.07* |
|  | Exposure to media: at least once a week | |  |  |  |  |  |  |
|  |  | Reading newspaper | 1.09 | (0.40, 2.95) | *0.87* |  |  |  |
|  |  | Listening to radio | 1.51 | (1.06, 2.16) | *** |  |  |  |
|  |  | Watching TV | 1.58 | (1.11, 2.26) | *** |  |  |  |
|  | Involved in decision making on | |  |  |  |  |  |  |
|  |  | How man's income is used | 0.95 | (0.67, 1.36) | *0.79* |  |  |  |
|  |  | Large household purchases | 0.99 | (0.71, 1.38) | *0.96* |  |  |  |
|  |  | Visiting family and friends | 1.18 | (0.85, 1.63) | *0.32* |  |  |  |
|  |  | Regarding own health care | 0.73 | (0.52, 1.03) | *0.07* |  |  |  |
|  | Appropriate attitude towards domestic violence: no queried situation was justified | | 0.80 | (0.50, 1.28) | *0.35* |  |  |  |
|  | Women's empowerment score (5 items) | |  |  |  |  |  |  |
|  |  | <Weighted mean | 1.00 | (Referent) |  |  |  |  |
|  |  | >=Weighted mean | 0.98 | (0.71, 1.36) | *0.92* |  |  |  |
| *Paternal characteristics* | | |  |  |  |  |  |  |
|  | Age (years) | |  |  |  |  |  |  |
|  |  | <25 | 1.19 | (0.73, 1.94) | *0.48* |  |  |  |
|  |  | 25-34 | 1.00 | (Referent) |  |  |  |  |
|  |  | >=35 | 1.23 | (0.78, 1.96) | *0.38* |  |  |  |
|  | Highest educational level | |  |  |  |  |  |  |
|  |  | No education | 1.00 | (Referent) |  | 1.00 | (Referent) |  |
|  |  | Primary | 1.55 | (0.95, 2.54) | *0.08* | 1.26 | (0.76, 2.09) | *0.37* |
|  |  | Secondary or higher | 2.09 | (1.45, 3.01) | **** | 1.28 | (0.85, 1.92) | *0.24* |
|  | Occupation | |  |  |  |  |  |  |
|  |  | Agricultural | 1.00 | (Referent) |  | 1.00 | (Referent) |  |
|  |  | Non-agricultural | 0.66 | (0.45, 0.98) | *** | 1.13 | (0.72, 1.78) | *0.59* |
| *Household characteristics* | | |  |  |  |  |  |  |
|  | Female household head | | 2.21 | (0.54, 9.16) | *0.27* |  |  |  |
|  | No. of HH members | |  |  |  |  |  |  |
|  |  | <Weighted mean (9.0) | 1.00 | (Referent) |  |  |  |  |
|  |  | >=Weighted mean (9.0) | 0.96 | (0.70, 1.32) | *0.80* |  |  |  |
|  | No. of children under 5 years | |  |  |  |  |  |  |
|  |  | <Weighted mean (2.3) | 1.00 | (Referent) |  |  |  |  |
|  |  | >=Weighted mean (2.3) | 0.92 | (0.67, 1.28) | *0.63* |  |  |  |
|  | Type of cooking fuel | |  |  |  |  |  |  |
|  |  | Electricity, LPG, natural gas, biogas | 1.31 | (0.90, 1.91) | *0.16* |  |  |  |
|  |  | Wood, straw/ shrubs/ grass, animal dung and other | 1.00 | (Referent) |  |  |  |  |
|  | Water source | |  |  |  |  |  |  |
|  |  | Unimproved source of drinking water | 0.47 | (0.33, 0.69) | **** |  |  |  |
|  |  | Source for water not in own dwelling or yard/plot | 0.78 | (0.55, 1.10) | *0.16* |  |  |  |
|  |  | Time to get to water source (min) |  |  |  |  |  |  |
|  |  | 0 | 1.00 | (Referent) |  | 1.00 | (Referent) |  |
|  |  | 1-59 | 0.75 | (0.53, 1.06) | *0.10* | 0.77 | (0.53, 1.13) | *0.19* |
|  |  | >=60 | 0.78 | (0.28, 2.12) | *0.62* | 0.91 | (0.31, 2.67) | *0.87* |
|  | | | | | | | | |
| *Supplemental Table 3 cont’* | | | | | | | | |
|  |  |  | Univariate association | | | Multivariate association | | |
|  |  |  | Estimate | | *P-value* | Estimate | | *P-value* |
|  |  |  | OR | (95%CI) |  | OR | (95%CI) |  |
|  | Toilet condition | |  |  |  |  |  |  |
|  |  | Unimproved toilet facility | 0.72 | (0.49, 1.05) | *0.09* |  |  |  |
|  |  | Shared toilet with other households | 1.40 | (0.99, 1.98) | *0.06* |  |  |  |
|  | HH wealth | |  |  |  |  |  |  |
|  |  | Richest | 1.00 | (Referent) |  | 1.00 | (Referent) |  |
|  |  | Richer | 0.54 | (0.33, 0.91) | *** | 0.55 | (0.32, 0.94) | *** |
|  |  | Middle | 0.49 | (0.29, 0.85) | *** | 0.68 | (0.37, 1.25) | *0.22* |
|  |  | Poorer | 0.82 | (0.48, 1.40) | *0.47* | 1.40 | (0.74, 2.63) | *0.30* |
|  |  | Poorest | 0.30 | (0.16, 0.58) | **** | 0.69 | (0.31, 1.53) | *0.36* |
| *Community characteristics* | | |  |  |  |  |  |  |
|  | Rural residence | | 0.55 | (0.35, 0.86) | **** |  |  |  |
|  | Geographical region | |  |  |  |  |  |  |
|  |  | Northern | 1.00 | (Referent) |  | 1.00 | (Referent) |  |
|  |  | North Eastern | 1.02 | (0.45, 2.31) | *0.96* | 0.95 | (0.41, 2.17) | *0.90* |
|  |  | Western | 0.37 | (0.17, 0.84) | *** | 0.32 | (0.14, 0.73) | **** |
|  |  | Central Highland | 0.43 | (0.12, 1.58) | *0.20* | 0.23 | (1.33, 0.00) | *0.08* |
|  |  | Capital | 1.46 | (0.71, 2.98) | *0.30* | 1.03 | (0.50, 2.16) | *0.93* |
|  |  | Southern | 0.44 | (0.20, 0.95) | *** | 0.62 | (0.25, 1.49) | *0.29* |
|  |  | South Eastern | 1.77 | (0.83, 3.79) | *0.14* | 1.77 | (0.76, 4.10) | *0.18* |
|  |  | Eastern | 0.93 | (0.43, 2.00) | *0.85* | 1.35 | (0.58, 3.14) | *0.49* |
|  | Women completed primary or higher education | | 4.37 | (2.03, 9.41) | **** |  |  |  |
|  | Women's empowerment | | 0.88 | (0.77, 1.00) | *** |  |  |  |
|  | Rank of access to health care | |  |  |  |  |  |  |
|  |  | Highest (best access) | 1.00 | (Referent) |  | 1.00 | (Referent) |  |
|  |  | Higher | 0.64 | (0.35, 1.16) | *0.14* | 0.70 | (0.38, 1.32) | *0.27* |
|  |  | Medium | 0.50 | (0.27, 0.92) | *** | 0.55 | (0.28, 1.09) | *0.09* |
|  |  | Lower | 0.49 | (0.27, 0.92) | *** | 0.61 | (0.28, 1.33) | *0.21* |
|  |  | Lowest (worse access) | 0.13 | (0.07, 0.26) | **** | 0.24 | (0.10, 0.59) | **** |
|  | Unimproved toilet | | 0.58 | (0.38, 0.88) | *** |  |  |  |
|  | Share toilet with other households | | 1.41 | (0.82, 2.42) | *0.22* |  |  |  |
