# Supplementary material for: Predictors of complementary feeding practices in Afghanistan: Analysis of the 2015 Demographic and Health Survey
Source: Matern Child Nutr. 2018 Nov 29;14(Suppl 4):e12696. doi: 10.1111/mcn.12696 (PMC6587761; doi:10.1111/mcn.12696)
Supplement: Supplementary file 2 — Table S4. Key demographic and socio‐economic predictors of complementary feeding practices by region in Afghanistan 2015. [file MCN-14-e12696-s002.docx]

| Supplemental Table 4: Key demographic and socio-economic predictors of complementary feeding practices by region in Afghanistan 2015 | | | | | | | | | | | | |
| --- | --- | --- | --- | --- | --- | --- | --- | --- | --- | --- | --- | --- |
|  |  |  | Northern | North Eastern | Western | Central Highland | Capital | Southern | South Eastern | Eastern | P-value from Chi-square test |  |
| **N** |  |  | 1593 | 1036 | 1048 | 183 | 1447 | 1357 | 571 | 702 |  |  |
| ***Child Characteristics*** | | |  |  |  |  |  |  |  |  |  |  |
|  | Still breastfed | | 84.4 | 82.9 | 75.2 | 89.3 | 74.4 | 81.9 | 69.3 | 82.6 | <0.0001 |  |
|  | Age (months) | |  |  |  |  |  |  |  |  | <0.0001 |  |
|  |  | 6-11 | 35.2 | 34.2 | 26.2 | 37.1 | 35.9 | 38.4 | 18.3 | 39.3 |  |  |
|  |  | 12-17 | 43.4 | 38.6 | 48.1 | 39.7 | 43.3 | 47.3 | 57.5 | 39.4 |  |  |
|  |  | 18-23 | 21.4 | 27.3 | 25.7 | 23.2 | 20.8 | 14.3 | 24.2 | 21.3 |  |  |
|  | Birth order | |  |  |  |  |  |  |  |  | <0.05 |  |
|  |  | Firstborn | 18.6 | 19.4 | 20.2 | 16.7 | 21.7 | 14.3 | 18.7 | 14.1 |  |  |
|  |  | Second to fourth | 47.3 | 42.5 | 45.6 | 45.1 | 46.8 | 52.1 | 49.7 | 41.4 |  |  |
|  |  | Fifth and more | 34.1 | 38.1 | 34.2 | 38.2 | 31.5 | 33.6 | 31.5 | 44.5 |  |  |
|  | Perceived birth weight | |  |  |  |  |  |  |  |  | <0.0001 |  |
|  |  | Smaller than average | 27.3 | 25.6 | 24.6 | 57.9 | 26.8 | 22.0 | 10.3 | 15.4 |  |  |
|  |  | Average | 63.6 | 53.2 | 63.3 | 34.4 | 55.2 | 63.9 | 72.8 | 66.6 |  |  |
|  |  | Larger than average | 9.1 | 21.2 | 12.1 | 7.6 | 18.0 | 14.1 | 16.8 | 18.0 |  |  |
|  | Complete age-appropriate vaccination | | 47.1 | 50.3 | 45.4 | 49.2 | 52.0 | 21.4 | 43.6 | 45.3 | <0.0001 |  |
| ***Maternal Characteristics*** | | |  |  |  |  |  |  |  |  |  |  |
|  | Antenatal clinic visits | |  |  |  |  |  |  |  |  | <0.0001 |  |
|  |  | None | 20.6 | 39.4 | 35.6 | 39.3 | 29.4 | 60.4 | 45.2 | 42.0 |  |  |
|  |  | 1-3 | 56.3 | 43.6 | 46.3 | 38.7 | 34.0 | 32.6 | 48.3 | 44.8 |  |  |
|  |  | ≥4 | 23.2 | 17.0 | 18.1 | 22.1 | 36.6 | 7.0 | 6.5 | 13.2 |  |  |
|  | Highest educational level | |  |  |  |  |  |  |  |  | <0.0001 |  |
|  |  | No education | 67.0 | 78.8 | 84.3 | 79.9 | 68.9 | 93.6 | 97.9 | 88.6 |  |  |
|  |  | Primary | 12.4 | 9.6 | 9.0 | 10.7 | 12.4 | 4.0 | 1.6 | 7.1 |  |  |
|  |  | Secondary or higher | 20.6 | 11.6 | 6.7 | 9.4 | 18.7 | 2.4 | 0.5 | 4.3 |  |  |
| ***Paternal Characteristics*** | | |  |  |  |  |  |  |  |  |  |  |
|  | Highest educational level | |  |  |  |  |  |  |  |  | <0.0001 |  |
|  |  | No education | 49.2 | 62.5 | 67.1 | 69.6 | 41.4 | 68.3 | 56.4 | 49.0 |  |  |
|  |  | Primary | 21.8 | 11.4 | 14.1 | 11.4 | 14.0 | 12.8 | 10.4 | 11.9 |  |  |
|  |  | Secondary or higher | 29.0 | 26.1 | 18.9 | 19.1 | 44.6 | 18.8 | 33.2 | 39.1 |  |  |
| ***Household Characteristics*** | | |  |  |  |  |  |  |  |  |  |  |
|  | HH wealth | |  |  |  |  |  |  |  |  | <0.0001 |  |
|  |  | Poorest | 26.1 | 33.6 | 39.5 | 60.1 | 4.3 | 1.4 | 4.7 | 4.3 |  |  |
|  |  | Poorer | 17.9 | 27.9 | 22.0 | 26.6 | 7.9 | 18.7 | 29.2 | 20.1 |  |  |
|  |  | Middle | 18.7 | 15.6 | 14.6 | 9.0 | 12.6 | 33.3 | 30.7 | 29.5 |  |  |
|  |  | Richer | 20.7 | 13.8 | 11.8 | 4.1 | 28.3 | 24.8 | 30.4 | 33.1 |  |  |
|  |  | Richest | 16.5 | 9.2 | 12.1 | 0.2 | 46.8 | 21.9 | 5.0 | 13.1 |  |  |
| ***Community characteristics*** | | |  |  |  |  |  |  |  |  |  |  |
|  | Rural residence | | 80.7 | 78.9 | 86.9 | 95.3 | 43.5 | 74.8 | 96.8 | 84.8 | <0.0001 |  |
|  | Average rank of access to health care | | |  |  |  |  |  |  |  | <0.0001 |  |
|  |  | Highest (best access) | 49.0 | 7.6 | 21.3 | 14.7 | 49.2 | 0.1 | 0.0 | 34.7 |  |  |
|  |  | Higher | 9.1 | 19.8 | 11.7 | 19.2 | 32.9 | 7.0 | 24.5 | 15.1 |  |  |
|  |  | Medium | 18.4 | 17.8 | 14.5 | 15.0 | 11.4 | 19.9 | 37.3 | 12.4 |  |  |
|  |  | Lower | 12.6 | 34.5 | 38.9 | 23.1 | 4.9 | 20.9 | 34.6 | 10.5 |  |  |
|  |  | Lowest (worse access) | 10.9 | 20.3 | 13.5 | 28.1 | 1.7 | 52.1 | 3.6 | 27.3 |  |  |
